# Supplementary material for: Holistic processing only? The role of the right fusiform face area in radiological expertise
Source: PLoS One. 2021 Sep 1;16(9):e0256849. doi: 10.1371/journal.pone.0256849 (PMC8409609; doi:10.1371/journal.pone.0256849)
Supplement: Attachment — Submitted filename: Response to reviewers.docx [file pone.0256849.s001.docx]

Response to reviews of *PONE-D-20-23315 Holistic processing only? The role of the fusiform face area in radiological expertise*

We would like to thank the Editor and the reviewers for the thoughtful comments and suggestions and the opportunity to revise and resubmit our manuscript. Below, we discuss the changes we made to the manuscript in response to each of the reviewers’ points. Line numbers refer to the manuscript file with tracked changes.

| **Comment** | **Response** |
| --- | --- |
| Thank you for submitting your manuscript to PLOS ONE. After careful consideration, we feel that it has merit but does not fully meet PLOS ONE’s publication criteria as it currently stands. Therefore, we invite you to submit a revised version of the manuscript that addresses the points raised during the review process.  There are a fair number of comments to be addressed by the Reviewers but they all seem highly pertinent to improve the overall quality of the manuscript. |  |
| In addition, please carefully proofread the manuscript as there are some typos throughout (e.g. "Them," instead of "Then," in the abstract). | This typo is now corrected, and we have conducted a careful proofread. |
| Also, I recognize that recruiting additional subjects for the study, as suggested, may not be feasible. However, please do your best to discuss this aspect within your manuscript. | Indeed, for practical and financial reasons, it is not possible for us to recruit additional subjects. We discuss the issue of sample size in the discussion (lines 544-556).  “A final important limitation of our study is the limited number of participants. Note, however, that it is hardly alone in this: Gauthier’s classic study on visual expertise in birds and cars found robust expertise effects in the FFA with groups of only eleven and eight participants [4], and in their study in which participants were trained to recognize Greebles, the ‘expert’ group consisted of only five participants. Recently, it has been argued that those earlier studies on the expertise account of the FFA with small sample sizes have overestimated the expertise effects [15]. At the same time, expertise research, in general, has a tradition of drawing rather small samples: Gegenfurtner et al. [21] report that eye-tracking research on expertise on average included 11 experts, 10 intermediates, and 12 novices per study (comparable to our sample). Behavioral effects are generally very large (e.g., none of the intermediates in our sample scored lower than the laypeople), so it was expected that effects of expertise on FFA activity would be large enough to find in a relatively small sample. Based on accumulating evidence, however, larger samples are increasingly recommended [15].” |
| Please also be careful about the use of the term "marginally significant". While you can discuss these results, the use of the term "trend" is more appropriate (e.g. lines 319-320). | We now use the term “trend” to discuss all effects with p-values between 0.1 and 0.05 (lines 369, 380, 386). |
| 1. Please ensure that your manuscript meets PLOS ONE's style requirements, including those for file naming. The PLOS ONE style templates can be found at  <https://journals.plos.org/plosone/s/file?id=wjVg/PLOSOne_formatting_sample_main_body.pdf> and  <https://journals.plos.org/plosone/s/file?id=ba62/PLOSOne_formatting_sample_title_authors_affiliations.pdf> | The manuscript is adapted to meet PLOS ONE’s style requirements. |
| 2.We note that you have indicated that data from this study are available upon request. PLOS only allows data to be available upon request if there are legal or ethical restrictions on sharing data publicly. For more information on unacceptable data access restrictions, please see <http://journals.plos.org/plosone/s/data-availability#loc-unacceptable-data-access-restrictions>.   In your revised cover letter, please address the following prompts:   a) If there are ethical or legal restrictions on sharing a de-identified data set, please explain them in detail (e.g., data contain potentially sensitive information, data are owned by a third-party organization, etc.) and who has imposed them (e.g., an ethics committee). Please also provide contact information for a data access committee, ethics committee, or other institutional body to which data requests may be sent.   b) If there are no restrictions, please upload the minimal anonymized data set necessary to replicate your study findings as either Supporting Information files or to a stable, public repository and provide us with the relevant URLs, DOIs, or accession numbers. For a list of acceptable repositories, please see <http://journals.plos.org/plosone/s/data-availability#loc-recommended-repositories>.   We will update your Data Availability statement on your behalf to reflect the information you provide. | We have updated the cover letter with the following information:  “We have an ethical restriction on sharing the de-identified raw data. Participants in our study were recruited from a small population. By publicly sharing raw data, individual participants could be identified by their colleagues. This is particularly problematic given the specific sample of radiologists in training: they have access to software to visualize the raw data and they have the ability to interpret those. This might make it possible to identify participants, which is considered problematic by the ethics committee, as participants might have felt pressured to participate. It was therefore considered unethical by the ethical committee to share raw data files publicly.  The SPSS file (without identifying information) that can be used to replicate the study’s results can be accessed on Dataverse, <https://doi.org/10.34894/O0CKVP>. .” |
| Reviewer #1: |  |
| The authors test whether the right FFA exhibits experience related responses to radiographs in radiology students vs a set of controls. The authors replicate the expertise-rFFA link like many papers before it, but only in the blocks where participants have to respond quickly. They do not find similar expertise related differences in V1, which the authors claim rejects the attention hypothesis of FFA expertise effects.  While it’s always reassuring to see a finding in the literature replicated, I think the authors could extend the literature in a more novel way by localising and examining these effects in other brain regions. | We would like to thank the reviewer for the positive response and thoughtful remarks.  The aim of our study was not only to replicate the expertise-rFFA link but also to understand the specific function of the FFA better (i.e. to support holistic processing) and to investigate the effect of expertise on FFA activation in a less experienced sample (residents). Although your suggestion to localize and analyze other regions is certainly interesting, it was not the original goal of the project and we do not feel it provides answers to the research questions that we formulated.  We have now reformulated the first sentence of the last paragraph and the first sentence of the discussion to make our goal more explicit:  “In the current study, we aim to investigate the specific function of the right FFA in visual expertise tasks in radiologists in training.” (lines 139-140)  “In the current study, we investigated the function of the right FFA in visual expertise tasks in radiologists in training.” (lines 437-439). |
| I also have some comments on the authors’ interpretations of their data, analysis and methodology. The study is low powered and the authors should acknowledge this or remedy it with more data (although I appreciate this latter solution may be impossible due to lack of funding). | It is indeed impossible for us to collect more data. As explained in response to the comments of the editor, we discuss the issue of sample size in the discussion (lines 544-556). |
| I should mention I am not an expert on the technical minutia of fMRI data recording or processing. This paper should therefore be reviewed by an fMRI expert too, one who can assess the technical aspects carefully. However, I will comment on these as best I can. Having read all fMRI expertise papers, I am much more familiar with the paradigms used, and the evidence that supports and rejects the expertise hypothesis, which I feel will help the authors in my review. |  |
| Major points  1. While replicating non-face FFA effects is interesting, it has now been shown in around 20-30 papers. One thing that is less clear is whether the left FFA or either OFAs are responsive to object expertise. When you examine the literature, it is only a tiny minority of papers that show effects in these regions (e.g., Harley et al., 2009; McGugin, Newton, Gore, & Gauthier, 2014; McGugin, Van Gulick, Tamber-Rosenau, Ross, & Gauthier, 2014; Ross et al., 2018). Why do the authors not try and remedy this by localising these other regions and exploring whether they are also responsive to expertise? Or, if they can’t, the authors should report why; i.e., maybe they can’t localise these regions, which has been mentioned in some papers. The same is also true of effects in the LOC too. | As explained above, the aim of this project was not to investigate the effects of expertise in regions other than the rFFA, since we aimed to further understand the function of the rFFA. The data collection was thus not optimized for investigating other brain regions and running other analyses, and those analyses would not align with the research goals. We feel that this is an important topic for further research and suggest this in the discussion: “Likewise, the holistic processing of stimuli is only one aspect of visual expertise, and it would be interesting to also investigate the involvement of other regions of the brain in other aspects of visual expertise.” (lines 519-521) |
| I note the authors mention that they only looked at the FFA to avoid employing multiple comparisons with other areas that would inflate the risk of a Type 2 error, but this is not a good reason to not perform these additional analyses. Burns, Arnold, & Bukach (2019, Footnote 4) found that conservatively speaking, expertise studies should test 56 participants even when engaging one-tailed analyses. That would place this study as underpowered with n = 28, or 17 experts (I should mention only one expertise study does not suffer this low power problem: Martens et al., 2018). It does not therefore make sense to perform corrections for multiple comparisons when power is low (Nakagawa, 2004). Instead, it would be more interesting to run the analyses on the other localised regions, and simply report that due to low power, the p-values will not be corrected. That way, we can see if there are expertise effects in other regions (which are not often reported in the literature outside of the rFFA). | We would like the thank the reviewer for suggesting to read the paper by Burns, et al (2019). which provides very relevant information on power and sample size, but was unfortunately published after our data collection.  We discuss the issue of sample size in the discussion (lines 544-556).  We also adapted the sentence in line 148 about the multiple testing to more clearly reflect that the use of a functional localizer avoids the multiple-testing problem (and not the choice to only include one functional localizer, which is funneled by our research question). It now reads:  “The use of functional ROIs provides more power to detect specific differences and, to some extent, avoids the multiple testing problem (Bennett et al. 2011).” |
| Also, were the reported analyses two-tailed? | We now added in line 386 that t-tests were one-sided.  “The one-sided t-tests”  We now also report this earlier, in line 317-318:  “In order to increase comparability with earlier literature [2, 18], two planned one-sided t-tests for the short-presentation trails were executed”  We additionally removed the line “For all other analyses, we used independent-sample t-tests (line 325-326), which might have caused confusion. |
| 2. Relatedly, how did the authors determine sample sizes? Did they analyse their data as each participant was recruited then stop when they found predicted results? Or was there an a priori number? It seems the sample size is similar to that suggested in the post 2010 Gauthier lab papers, but the authors do not specify this. | We now added that: “An a-priori determined sample size of 10 residents in their first year and 10 residents in year 3 or higher was not met for practical reasons so these were combined into one group (radiologists in training). (lines 168-170). |
| 3. The authors say there is a need for a non-passive study, but this doesn’t sound right. When I double checked, citation 2 (Harley et al) already did a diagnostic fMRI task in radiologists. While their localizer scan was passive, which is common, in the actual experiment the participants tried to identify the nodules (from the paper follows): “The diagnosis scans were event-related scans in which participants viewed intact and coarsely scrambled radiographs and judged whether a cued region in each radiograph contained a lung nodule” This means what the authors say has not been done, actually has been. The more novel aspect of their study therefore is the 2s vs 10s difference. I think the authors may want to reread their other citations to make sure there aren’t other errors when describing the literature. | We thank the reviewer for pointing us to this flaw. We have now adapted the text to better reflect the literature and to stress our focus on the short vs. long presentation time (lines 126-137).  “Two of the neuroscientific studies that investigated radiological expertise so far aimed to capture the participants’ processing of stimuli, but not specifically the diagnostic processes. They thus asked participants to execute a 1-back task [18] or a manipulation detection task [25]. In studies that did require participants to detect or diagnose abnormalities, radiographs were presented for relatively short amounts of time only, such as 500 ms [2], or 1500 ms. [26]. Tasks aimed at processing but not diagnosing radiographs, and very short tasks are likely to elicit a holistic but not a search-to-find mode. If the FFA plays a crucial role in the process of holistic perception, it is more likely to differentiate between expertise levels if radiographs are observed for short periods of time and less so if participants engage in the slower search-to-find mode. Thus, there is a need for research that investigates the activation of the FFA during longer presentation periods to contrast this with FFA activation during shorter task durations, to better understand the specific function of the FFA in the diagnostic process..”  We also added similar information in the discussion (lines 492-496):  “However, two of those studies [18, 25] employed tasks that were not tapping into the process of diagnosing radiographs, but instead employed unrelated tasks that required participants to only look at radiographs. Harley and colleagues [2] required participants to detect the presence or absence of cued tumors, and only Melo and colleagues [26] required participants to formulate a diagnosis (but for shortly presented radiographs only).” |
| 4. I can’t think of a single fMRI expertise study that has made their raw data publically available even though there are repositories available for this very purpose (D’Esposito, 2000). Why do the authors not make their data available? It would help start a precedent, improve transparency and replicability, and future researchers could reuse the data with other sets to increase power. Or do the authors have a particular reason for not sharing their data? | In our situation, we have a strong ethical reason not to share publicly: participants in our study were recruited from a small population. By publicly sharing raw data, individual participants could be identified by their colleagues. This is particularly problematic given the specific sample of radiology residents: they have access to software to visualize the raw data and they have the ability to interpret those. It was therefore considered unethical by the local ethical committee to share those files publicly. Researchers without a relationship with the participants can receive the raw data upon request. |
| 5. Regarding the correlations between expertise and neural activity, I think it would help if they were actually plotted (they could go into sup matts if the authors prefer). That way the reader could see individual data points. | We thank the reviewer for this excellent suggestion. We replaced figures 4 and 5 with plots of individual beta values against score with expertise color-coded. |
| Also, a table of the within group and across groups correlation co-efficients and p-values within each condition/location would also be helpful. Gauthier and colleagues in their most recent papers recruit participants with a broad range of expertise (i.e., they’re not all experts) and find correlations between activity and FFA activity, so having all participants analysed together would help increase power. | The participants in our laypeople group did not have any expertise in radiology. Since we implemented a forced-choice test to measure performance, we obtained scores from participants that are above zero, but that reflect that the participants are just guessing. Including those scores in the correlation would bias the actual correlation in participants with at least some expertise. We therefore only include the correlations within the group of radiologists in training (see lines 423-427). The pattern of results in the full group can be appreciated in the new figures 4 and 5. |
| 6. It is common for researchers to find the voxel in the FFA that activates the most to faces, and then test whether this is also the peak for expertise, did the authors do this here? I can’t see mention of it. | We chose the established and widely applied region-of-interest (ROI) analysis (ROI) approach. This approach has the advantage that it is more robust for motion than the peak-voxel approach. If a participant would shift only slightly, a one-voxel FFA would very likely to be no longer overlapping with the selected voxel, whereas a slight shift would still cause a larger portion of the FFA to overlap with the chosen region of interest when the ROI approach is used. |
| Minor points  Page 3 line 60, I’m not sure there is a general consensus. I still meet countless people at conferences/talks who claim the expertise hypothesis is not real. This is reflected in the literature where Kanwisher (2017) still argues against these effects, with echoes of this present in another recent review (Duchaine & Yovel, 2015). | We have adapted the wording to reflect the issue raised by the reviewer (lines 60-63).  “Although some researchers still argue that the FFA is uniquely dedicated to faces [8-10], there is now ample evidence to suggest that the FFA plays a crucial role not only in face perception but more broadly in visual expertise [e.g., 3, 4, 11-15], as voiced in the general expertise hypothesis.” |
| The authors touch upon prosopagnosia, so it may be worth noting that there are three separate studies from Jason Barton’s lab of groups of patients with FFA lesions that exhibit impaired object expertise (the citations are in the recent Burns et al 2019 expertise meta-analysis paper). One of these papers doesn’t specify the patients have FFA lesions, but if you check their earlier papers, you can identify that the cases actually do. I think when combined with the fMRI data, the expertise hypothesis becomes very compelling. In the intro the authors ask what the FFA is doing in expertise, and I think this data strongly suggests it contributes to the experience based individuation of highly similar object exemplars. | We have added work from Jason Barton’s lab in our argumentation (lines 67-73).  “In participants with FFA lesions resulting in prosopagnosia, high expertise in car recognition as measured with a verbal test did not result in an equally high ability to visually recognize cars, whereas those two variables were highly correlated in healthy controls. This suggests that patients with prosopagnosia also have trouble visually individuating highly similar objects (e.g., recognizing the model, manufacturer, and decade of make of cars) [17], and, likewise, that the FFA plays a role in this visual individuation of highly similar stimuli.” |
| The authors refer to chest pieces, but I think they mean chess. | We corrected this typo in line 76. |
| Page 4 The authors don’t really define holistic processing (or many other terms they introduce). What is it? Does inversion disrupt it because we are no longer using experience based pathways which would typically recognise such objects in its commonly seen upright configuration? Where’s the evidence that speed is a more central feature of holistic perception than stimulus configuration? What is search to find? What are the conceptual similarities pointed out by Bilalic? It’s not explained clearly why short versus long duration should differentiate experts in their neural activity? | We have rephrased a section of page 4 to give a clearer account of the visual expertise literature and its definition of holistic processing, and also explain (rather than pose) that speed is central in this conceptualization, and why the short versus long duration should differentiate those processes. (lines 89-107).  “Holistic processing is a central aspect of theories of visual expertise in general, and visual expertise in radiology in particular [20]. Visual-expertise research in radiology typically assumes a two-phase diagnostic process, consisting of a first, relatively fast, ‘holistic mode’ followed by a slower, ‘search-to-find’ or ‘checking’ mode [5]. The holistic mode entails an initial global analysis of the entire retinal image to distinguish normal from abnormal tissue, which subsequently guides search to perturbations using foveal vision (the checking mode) [5]. The global impression is a comparison of the contents of the radiograph to an expert’s schema of the visual appearance of normal radiographs. Central in this conceptualization of holistic processing is speed [20-22]: This global impression is developed first in the diagnostic process, and visual experts have been found to develop a global impression or gist of an image within 250 – 2000 ms [1] or less [5, 23, 24]. The slower ‘checking’ mode is more feature-based and involves shifting selective attention to potentially relevant areas of radiographs. Given that the FFA is mostly linked to the holistic processing of faces and objects of expertise, it seems likely that the FFA is less involved in the feature-based checking-mode that generally takes place after the initial holistic mode. However, investigating if this is indeed the case is only possible in experimental tasks that elicit the full diagnostic process and separates those phases. Complete separation of holistic and checking modes is not possible, but we argue that the short presentations of radiographs mostly elicits the holistic mode (since there is no time to enter the checking mode), whereas a longer presentation time in combination with the instruction to check an earlier diagnosis is expected to elicit mostly the checking mode.” |
| Why is the FFA the most theoretically relevant? How do we know it’s the most relevant if the other regions (e.g., OFA, lFFA) have not been not as well studied? | The research questions asks for the function of the rFFA, which means that the analysis of the OFA and the lFFA would not fit the aim of the study.  We have rephrased this sentence, and it now reads “However, the use of a localizer task for the FFA allows us to focus our analysis on the function FFA.”(lines 146-148). |
| Page 14 I presume the authors look at V1 because others have suggested FFA expertise activity is an artefact of attention, however, the authors do not explain this, nor cite the previous literature positing this (Harel et al, 2010; Kanwisher, 2017). Prior work has tried to address this issue (McGugin et al., 2015; McGugin et al., 2016) and there are other problems with this hypothesis (Burns et al., 2019). | We would like to thank the reviewer again for drawing our attention to the work of Burns et al., and in line with their argumentation, we have decided to remove the V1 analyses from the paper completely. |
| Change level should be chance level? | We corrected this typo in line 343. |
| Page 16 Marginally significant effect was not significant and so should be reported as such (p = .052). Nor is the later interaction significant (p = .08). Despite this latter result, I’m guessing the subsidiary analyses were preplanned based upon the authors’ hypotheses and prior literature, hence the motivation to perform them despite the interaction not being significant? If so, this should be explained. | As recommended by the editor, we now discuss all p’s between 0.1 and 0.05 as ‘trends’.  Furthermore, we now write that “In order to increase comparability with earlier literature [2, 17], two planned one-sided *t*-tests for the short-presentation trails were executed: one for correctly diagnosed trials only and one for all trials” (lines 317-319) |
| When performing the correlations between FFA activity and performance, would it not be interesting to partial out the V1 activation? If the authors argue this activity is attention related, then taking it into account may yield a non-attention related relationship between FFA and expertise? The authors could note these were exploratory as they were not planned. The logic behind this type of analysis is detailed in DeGutis, Wilmer, Mercado & Cohen (2013) and has been used in many of the McGugin FFA expertise papers. While they regressed out different types of behavioural performance, and I haven't seen it used to regress out brain activity, I believe it the same logic could be applied and be useful here. | In line with the argumentation of Burns et al., that you mentioned earlier, the V1 activation should not be taken as an indication of engagement with the task, so following that logic, regressing out V1 activation in the FFA activation is not tried. |
| The correlation coefficients of around .2 are actually at the most conservative end of the predicted expertise-FFA relationships when the broad literature is taken into account (Burns et al., 2019; Footnote 4). Again hinting the effect may be there, it’s just that insufficient numbers were tested. As mentioned earlier, why not run the correlations across groups (and plotted), as it would increase power and give a broader range of expertise. | As mentioned above, we ran the correlation only in the group of radiologists in training but show the data for all participants in figures 4 and 5. |
| I find it strange the authors did not compute dprime as this was what was correlated with the FFA in the Harley paper. | The measure d-prime is implicated in situations where a target is present or absent, so for each participant, there is a hit rate (target present and classified as present) and a false alarm rate (target absent but classified as present). It is thus mostly relevant if the task is to detect abnormalities.  In our stimuli, the task was not to detect but to diagnose or classify abnormalities. Hit rate and false-alarm rate can thus not be calculated as no target-absent trials are included. |
| Page 20  A simpler explanation for the different pattern of significant results in this and the Harley paper is that both were underpowered (Burns et al., 2019). If both studies only have 50% power and run two different analyses testing for a real effect, then it’s not surprising if only one of them is significant and the other not. If power was extremely high (>99%), we would expect both to almost always be significant. This in my mind is the most likely explanation for why analyses on the link between FFA and expertise can be inconsistent even within a paper, with authors giving quite complicated explanations for incongruent results (and this occurs in almost all expertise papers). Instead, low power is the simplest and most likely explanation. | We now discuss low power also as a limitation in the discussion (lines 544-556). However, looking at the beta’s in figures 4 and 5, the pattern of results matches our interpretation that the FFA is mostly active during the holistic mode and less so during the checking mode. |
| Page 21 The Haller and Melo papers did not test the FFA (i.e., no localiser task) so it is not accurate to report this as such. | We now added that these papers did not employ a localizer task (lines 489-491).  “Both Haller and Radue [25], and Melo and colleagues [26] did not report areas with significant activation that overlap with the FFA (note that they did not employ an FFA localizer task but only report areas with significant activation)” |
| Reviewer #2: The authors described their work on an interesting question that whether FFA is responsible for only holistic processing of expertise images but not for slower processing mode, which is a new angle lacks research. They have used a new set of task that required the subjects to do a diagnose based on a picture, which is better representing the real-life processing of the images, which engages attention, serial search and other reasoning.  The results seemed solid, only with big variations between subjects. Error bars are huge. Recruiting more subjects, getting rid of the subjects with big movement artifact would largely improve that. The correlation analysis may also improve if more subjects included. | We would like to thank the reviewer for the positive response and thoughtful remarks. As discussed in response to the comments of the editor, we cannot recruit more participants.  We did not include participants with considerable movement artifacts in our data analysis. To clarify how we checked for that, we added the following text in lines 282-284:  “After 3D motion-correction was executed, the motion correction parameters were plotted and visually inspected. No runs had to be discarded for excessive, non-correctable motion.” |
| It’s also good if they can show individual beta values in figure 4. | We have now replaced figure 4 with figures 4 and 5 that show individual beta values. |
| There is one issue with the design though. As is understandable that it is always more difficult to claim a negative conclusion. The authors found FFA response in expert group is lower during 10s task than 2s task. They contributed such difference into FFA’s selectivity to short presentation. However, such difference could also due to: 1. Novelty effect/task difficulty. All pictures in 10s task were already seen in 2s task. Therefore less engaged, worse attention. 2. Longer exposure of the same image adapted the visual system, and BOLD signal became weaker. Such general mechanisms could contribute to the difference. And the V1 response also showed similar trend – 10s trials showed lower response than 2s trials, which again indicated such difference is not a unique nature of FFA processing, but could be contributed to the task or BOLD signatures. | We now discuss our reasons for this design choice and its corresponding limitations in more detail in lines 522-543:  “Another limitation related to this aim is that participants were required to check their diagnosis based on the short-presentation runs during the long-presentation runs to elicit the checking mode. Thus, the order of short- and long-presentation runs could not be counterbalanced and we could not use a new set of images in the long-presentation runs. This could have caused order effects and/or effects of novelty. That is, all pictures in the long-presentation runs were already inspected in the short-presentation runs which could have caused disengagement. Not only was the repetition of stimuli necessary to elicit the checking mode, by using the same stimuli in the long-presentation runs and short-presentation runs, we also ensured that the difficulty of the two modes was the same. Furthermore, it has to be noted that although the images were presented twice, even a presentation time of 10 seconds is shorter than what residents would normally spend on an image, making disengagement an unlikely explanation of the lower activity during the longer trials. Anecdotally, participants remarked that they would have preferred seeing the images even longer. Finally, it has to be noted that we do not interpret the main effect of trial length, but only the main effect of expertise and the interaction of expertise and trial length. Even so, further research could counterbalance the order of the short-presentation and long-presentation runs, to entirely exclude a possible order effect. This would also require equally difficult stimuli in the two modes and an accordingly adapted instruction in the long-presentation runs.” |
| If the authors could make the 10s task more challenging, I doubt FFA may have bigger response even with a slow processing mode. For example, if the 10s task use new set of images (not seen ones), and the subjects need to find the abnormalities as many as possible (not a yes or no question), or it's a new set of more difficult images (rather than easy typical educational examples), the subjects may engage more in the task. | We now explain why we did not use new images in lines 522-525 “participants were required to check their diagnosis based on the short-presentation runs during the long-presentation runs to elicit the checking mode. Thus, the order of short- and long-presentation runs could not be counterbalanced and we could not use a new set of images in the long-presentation runs.”  We also explain in lines 222-224 that the yes/no format is actually aimed to capture diagnostic reasoning. “The yes/no format was used because it eliminated the need for participants to speak, which can cause motion artifacts that deteriorate fMRI data quality. At the same time, by requesting participants to engage in diagnostic reasoning while the image was on the screen, we capture diagnostic reasoning processes and not just perceptual processing.”    Furthermore, in response to your previous comment, we discuss why we do not think that (dis)engagement with the tasks is likely to have caused the effect, see lines 533-536.  “Furthermore, it has to be noted that although the images were presented twice, even a presentation time of 10 seconds is shorter than what residents would normally spend on an image, making disengagement an unlikely explanation of the lower activity during the longer trials. Anecdotally, participants remarked that they would have preferred seeing the images even longer.” |
| The author didn’t mentioned whether they tracked the eye position or not, nor did they mention whether the subjects are required to fixate or free viewing. I assume that the subjects will make more saccades during the 10s task, and that may cause more variations in to the overall signal. | In line 226-227 we added that “Participants were allowed to free-view the images, eye positions were not tracked.” |
| Other typos. Line 68, 69: chess, not chest Line 315: Figure 4 (right) | We corrected the typo in line 76. With the change of the figures, the typo in line 315 no longer present. |
