# Supplementary material for: Holistic processing only? The role of the right fusiform face area in radiological expertise
Source: PLoS One. 2021 Sep 1;16(9):e0256849. doi: 10.1371/journal.pone.0256849 (PMC8409609; doi:10.1371/journal.pone.0256849)
Supplement: Attachment — Submitted filename: PLOSONE 2.docx [file pone.0256849.s002.docx]

The authors have edited their manuscript to correct some issues, but some of their responses need further clarification, while other haven’t addressed my original comments. I realise my major points are quite long this time, but this is just because I want to ensure I’m clear enough so that we avoid any misinterpretations. I hope the authors don’t think I’m being intentionally difficult or disingenuous, it certainly isn’t my intention. I just want to ensure the decisions the authors have made in their methods and analyses are transparent to future readers, and that there is not a risk of readers misinterpreting how the literature currently stands with respect to power considerations.

Major points

1. The authors state that they can’t look at the lFFA or OFAs because they never intended to study them in the first place. Yet they have now removed a priori research questions. See the authors’ two responses to reviewers:

*“Although your suggestion to localize and analyze other regions is certainly interesting, it was not the original goal of the project”*

*“in line with their argumentation, we have decided to remove the V1 analyses from the paper completely.”*

I agree it’s perfectly fine to focus your manuscript on what the original research questions were, and these should be reported as a priori hypotheses. What is problematic is that these claims are at odds with the authors removing their original V1 hypotheses and analyses after the first review. These have completely vanished, as if they never existed. Yet this was exactly what the authors planned. The replication crisis has shown us that removing or altering hypotheses post-hoc to fit the data promotes false positives (seems a form of HARKing, Kerr, 1998, where authors drop a priori hypotheses). At the most fundamental level, the V1 data must be reinstated in the manuscript.

Following this train of thought, why can’t they at least attempt analyses on the lFFA, and/or the OFAs, and report that these as exploratory (or report an inability to localise). You’ve spent a lot of time, effort and expense acquiring this data, so why not report it (it would also add to the novelty, provide effect sizes or illustrate localisation issues which are common in the literature for other researchers)? Especially when the raw data are not immediately available for others to answer these questions themselves.

I’m not trying to be difficult, but these competing arguments over original research questions and post-hoc questions do not accord with one another.

2. I don’t think it will affect the importance of the manuscript if the V1 data is linked to the FFA effect, it is better to be transparent so readers can make up their own minds. This is why performing correlations between V1 and FFA activity are interesting (same is true with the partial out method). As I mentioned previously, even if there is a link between V1 and FFA effects, this is still consistent with the expertise hypothesis: see Gauthier’s recent commentary on Kanwisher’s paper on the history of FFA research, and Lohse et al. (2016) on this link to face regions. If the face processing link is expertise based, it makes sense if there’s a link due to non-face expertise. You should report these as exploratory.

3. Can’t the authors ‘deface’ the raw fMRI data so they remove the risk of identification? Doesn’t this mean they could then make the data publically available? Also, I can’t access the data file the authors provide to replicate their analyses.

4. The description in the Methods of how the authors decided their sample size still doesn’t appear to be based on any objective criteria. Saying you decided to test 10 in this group and 10 in another group is not the same as using power estimates from prior work to decide sample sizes. I am mindful that pushing this issue further may make a posthoc decision appear, but why were these numbers decided (the authors mention line numbers 168-170, but I don’t see this in my pdf, maybe they mean 147-149), was power ever considered when designing your study? The decision to stop at 17 experts for practical reasons is again too vague. What were these reasons? Funding spent elsewhere? Funding expiring? Scanner was no longer available to the researchers? You asked all your radiologists and the others said no? Or you stopped as you found a significant effect? It should be stated.

5. The following points all touch upon issues of power that the authors discuss.

The authors cite Gegenfurtner et al. in their discussion, which is a review of eye tracking effect sizes, but the current study is fMRI. I don’t think this is appropriate and should be removed as it reminds me of the Cow-Canary problem where effects can be massively different between two different methods (Capitani et al., 1999). The authors’ own behavioural data here shows a huge effect size (which would be expected between experts and novices), yet only a medium one in the fMRI data. Why discuss eye-tracking effect sizes when you had 20-30 fMRI expertise studies to base your decision on, it doesn’t make any sense in this context and should be removed as just confuses the issue.

In the same place in the Discussion the authors seem tacitly argue two fMRI expertise studies that are 20 years old are an acceptable guide for sample sizes (Gauthier et al., 1999: which they don’t cite here but describe, and Gauthier et al., 2000). This ignores the recent studies that used larger sample sizes (peaking with the recent Martens et al paper, which is not cited, nor most other studies in the last 10 years). Even if two 20 year old papers were the basis for making their sample size decisions, it should be mentioned in the Methods and not the Discussion (although again, a question remains as to why the authors selected such old papers to base their sample sizes on rather than more recent work).

From the above I hope it’s clear why the current Discussion paragraph on effect sizes from eye tracking and two 20 year old fMRI papers does not work. I don’t want future researchers reading this paper to think these sample sizes would lead to sufficient levels of power when designing their own fMRI expertise studies. I think it would be more helpful to explicitly remind the readers that we now know this study and Harley’s were likely underpowered, that classic papers from 20 years ago were underpowered, and that significant effects here were found using liberal alphas. It’s perfectly fine accepting the limitations of this manuscript, but they need explained to the readers so they’re aware of the issues.

6. Also related to the previous points. Maybe I was not clear enough in my first review, but when I referred to page 20 and the different patterns of results, I meant that you failed to find a correlation but Harley and colleagues did. However, you found a group experience difference that Harley didn’t. The simplest explanation for this disparity is that both studies are underpowered. If you have two studies that are underpowered (maybe 50ish%), then it’s likely one (Harley et al) will find a significant effect in one analysis, but not the other, while the second paper (the current manuscript) finds the opposite pattern of significant/non-significant results with the same analyses. You haven’t discussed this in this paragraph, nor in the later paragraph where you argue your study is not underpowered based on two studies from 20 years ago. You should explain this hypothesis of the conflicting results explicitly when discussing the differences between Harley and yourselves.

I should add, the discussion of the 2s vs 10s trials is distinct from the point I’m trying to make here (although a similar logic could be applied, I think the correlation/group difference disparity needs explained separately).

Minor points

1. The authors uploaded two different versions of their manuscript, one with tracked changes indicated and another without, but switched between them when they were referring in their response to reviewers (and they sometimes referred to line numbers that didn’t correspond with the text). This made checking between the different texts confusing and overly time consuming to review (and apologies if I’ve now referred to one text and the other). I’d recommend on the next submission they simply change the font color of the text they’d edited and upload a single manuscript (unless this is a journal specific requirement to upload two?). Same is true of having the figure legends next to the actual figures.

2. I think the authors have misinterpreted signal detection theory by claiming they can’t compute dprime. They employed a 2AFC task, with stimulus pairs that were same or different. This leads to categories of hits, misses, CRs and FAs. It’s quite easy to compute dprime to test whether this behavioural measure is more strongly correlated to FFA activity as it was in Harley et al. I’m convinced other readers would want to see this to compare the results between the current study and Harley et al.

3. Line 84: “processing a face as a whole, and not as a combination of features”

I think referring to ‘combination of features’ implies a holistic relational percept. Maybe something like “as separate, distinct features that do not interact to form a single percept” is clearer?

4. Removing the original graphs stops making the group differences/similarities in the different conditions easily comparable so should be reinstated. I like the individual plots, but trendlines would be a useful addition too.

5. Line 490 still reads like prior studies analysed an area that included the FFA (i.e., you mention overlap), but we can’t say this as they did not localise it. It could be in the vicinity of the FFA, but not actually overlap . Related to this, there are many expertise FFA studies that did localise the FFA and are not cited in this manuscript which seem more relevant (particularly the Martens et al study which should be the gold standard going forward). Surely at least from a point of generating interest in the current paper the authors would want to reference these as more citations lead to more exposure?
