# Supplementary material for: Holistic processing only? The role of the right fusiform face area in radiological expertise
Source: PLoS One. 2021 Sep 1;16(9):e0256849. doi: 10.1371/journal.pone.0256849 (PMC8409609; doi:10.1371/journal.pone.0256849)
Supplement: Attachment — Submitted filename: Response to reviewers.docx [file pone.0256849.s003.docx]

| Editor |  |
| --- | --- |
| Upon review of the revised version, the Reviewers and myself continue to have concerns about the current manuscript. I agree with both Reviewers that the V1 results should not have been removed from the revised manuscript. Please pay careful attention to respond to each of the points that have been raised. Although there is a non-negligible amount of work to do, I trust that the detailed reviews and feedback from the Reviewers will help facilitate your revision. | Thank you for the detailed feedback and the opportunity to revise the manuscript. We have now reinstated the V1 analyses and have responded below to all comments of the reviewers. |
| Reviewer #1: |  |
| The authors have edited their manuscript to correct some issues, but some of their responses need further clarification, while other haven’t addressed my original comments. I realise my major points are quite long this time, but this is just because I want to ensure I’m clear enough so that we avoid any misinterpretations. I hope the authors don’t think I’m being intentionally difficult or disingenuous, it certainly isn’t my intention. I just want to ensure the decisions the authors have made in their methods and analyses are transparent to future readers, and that there is not a risk of readers misinterpreting how the literature currently stands with respect to power considerations. | Thank you for the detailed feedback, you can find our responses below. Page numbers and line numbers refer to the version with ‘track changes’. |
| Major points 1. The authors state that they can’t look at the lFFA or OFAs because they never intended to study them in the first place. Yet they have now removed a priori research questions. See the authors’ two responses to reviewers:  “Although your suggestion to localize and analyze other regions is certainly interesting, it was not the original goal of the project” “in line with their argumentation, we have decided to remove the V1 analyses from the paper completely.”  I agree it’s perfectly fine to focus your manuscript on what the original research questions were, and these should be reported as a priori hypotheses. What is problematic is that these claims are at odds with the authors removing their original V1 hypotheses and analyses after the first review. These have completely vanished, as if they never existed. Yet this was exactly what the authors planned. The replication crisis has shown us that removing or altering hypotheses post-hoc to fit the data promotes false positives (seems a form of HARKing, Kerr, 1998, where authors drop a priori hypotheses). At the most fundamental level, the V1 data must be reinstated in the manuscript. | *We agree that removing a priori hypotheses is not recommended research behavior. Our intention with this investigation was to contribute to the literature by investigating the rFFA, and that is what we optimized the study for. Our a priori hypotheses concerned the rFFA only. We originally added the V1 analyses based on reviewer comments on an earlier draft. We do not consider them (and do not present them) as a priori hypotheses but rather as control analyses. However, after your first review, we felt in doubt about the argumentation and the validity of those analyses, and based on your comments and the Burns et al paper, we felt the arguments for removing them were stronger than those for keeping them in.*  *Based on your comments and those of the other reviewer, we have now reinstated the V1 analyses as control analyses in the manuscript.*  *See page 6 lines 138-140:* “Apart from localization of the right FFA, we use similar procedures to localize the right V1 to rule out attention effects.”  *Page 17 lines 331-336*: “As a check that the pattern of results was not already reflected in early visual cortex and therewith might result from a different amount of attention paid to the stimuli (e.g., experts might pay more attention to the stimuli because they are relevant for them), we repeated the analyses for hypothesis 2 in the V1 ROI. Also, on reviewer request, we conducted ANCOVAs with V1 activity as covariate, right FFA activity as dependent variable and expertise (laypeople and radiologists in training) for each of the repeated measures.”  *Pages 21-22, lines 432-44:* “**Analyses of V1 activity**  To exclude overall activation differences (already observable in early visual cortex) between the short- and long-presentation trials, we repeated the analyses in the right V1 ROI. A 2x2 ANOVA was run with factors expertise level (laypeople vs. radiologists in training) and trial length (2s trials vs. 10s trials) and V1 activity as the dependent variable (see Fig. 6).  **Fig 6. Density plots (upper panels) and scatter plots (lower panels) for the individual beta values in the right V1 for short- and long-presentation trials.** In the upper panels, the dotted line denotes the group mean. In the lower panels, for residents, darker colors depict more experienced residents.  The interaction of expertise with trial length was not significant, *F*(1,26) = 1.020, *p* = 0.32, *η^2^_p_* = 0.038 (small effect size). There was a significant effect of trial length, *F(*1,26) = 15.821, *p* < .01, *η^2^_p_* = 0.378 (large effect size), with lower betas for the long-presentation trials. There was no significant effect of expertise level, *F*(1,26) = 0.581, *p* = .45, *η^2^_p_* = 0.022 (small effect size).  We additionally analyzed the mean beta value in the V1 for the correctly diagnosed trials only (See figure 7). There was no significant interaction, *F*(1,26) = 1.614, *p* = .22, *η^2^_p_* = 0.058 (small effect size), a main effect of trial length, *F*(1,26) = 23.752, *p* < .01, *η^2^_p_* = 0.477 (large effect size), and no main effect of expertise level, *F*(1,26) = 1.128, *p* =.30, *η^2^_p_* = 0.041 (small effect size).”  *We added a paragraph in the discussion about the V1 analyses, pages 25-26 lines 507-521:* “The analyses of V1 activity provide an insight into the attention account of expertise effect, which holds that expertise effects in the FFA and other brain regions are simply the effect of greater attentional engagement with the objects of expertise [9, 30]. That is, an overall larger level of attention to objects of expertise by experts (because they are more interested in those stimuli) causes expertise effects not just in the right FFA but also in other regions of the visual system, such as V1. Gauthier argues against this account, showing expertise effects in the right FFA even with limited attention (e.g., when the object of expertise is irrelevant), and showing expertise effects even in the regional grey matter thickness of the right FFA. To explore the attention account of expertise for our data, we investigated whether radiologists in training show larger V1 activation (as a result of larger attentional engagement) then novices, and found no evidence for this. Additionally, as suggested by reviewers, we investigated whether partialling out V1 activation removes expertise effects, and found indeed that differences between conditions were no longer significant after partialling out V1 activity. This means that we cannot exclude that increased attention for the stimuli by residents compared to laypeople might explain the pattern of results in the right FFA. On the other hand, the V1 activity is not a significant covariate in any of the ANCOVAs. “ |
| Following this train of thought, why can’t they at least attempt analyses on the lFFA, and/or the OFAs, and report that these as exploratory (or report an inability to localise). You’ve spent a lot of time, effort and expense acquiring this data, so why not report it (it would also add to the novelty, provide effect sizes or illustrate localisation issues which are common in the literature for other researchers)? Especially when the raw data are not immediately available for others to answer these questions themselves. I’m not trying to be difficult, but these competing arguments over original research questions and post-hoc questions do not accord with one another. | *First of all, we would like to stress that the raw data are available for other researchers on request, and that the data manager will only deny access to participants of this study and their direct colleagues.*  *As pointed out by the reviewer, we had stayed too close to currently availably literature, and hence we consider it useful to add an analysis of the lFFA. Therefore, we have added the analysis of the lFFA and included the lFFA in figures 4 and 5.*  *Page 6-7 line 139-140*: “Finally, as an exploratory analysis, we investigated the lateralization of the expertise effect.”  *Page 14, lines 291-294:* “Additionally, we defined individual ROIs for the right primary visual cortex (V1). These ROIs were defined by a conjunction analysis (faces vs. resting and objects vs. resting). The most significant voxel in V1 was determined and subsequently, less stringent p-values were chosen until the ROI encompassed approximately 100 voxels (varying from 104 to 112 voxels).”  *Page 16, lines 309-314:* “Next, as requested by reviewers, we also ran an exploratory 2x2x2 ANOVA with factors expertise level (laypeople and radiologists in training), trial length (2s trials and 10s trials), and location (left FFA and right FFA) and beta value as the dependent variable. Those ANOVAs were followed up by post-hoc t-tests with factor expertise (laypeople and radiologists in training) with Cohen’s d as an effect size, where 0.2 denotes a small, 0.5 a medium, and 0.8 a large effect [29].”  Page 19-20 lines 384-397: “We also ran an exploratory 2x2x2 ANOVA with factors expertise level (laypeople and radiologists in training), trial length (2s trials and 10s trials), and location (left FFA and right FFA) and beta value as the dependent variable. The three-way interaction between expertise level, trial length and location was significant, *F*(1,26) = 4.395, *p* = .046, *η^2^_p_* = 0.145 (medium effect size). Furthermore, the two-way interaction between location and expertise level showed a trend towards significance, *F*(1,26) = 3.478, *p* = .07, *η^2^_p_* = 0.118 (medium effect size). The two-way interactions between trial length and expertise, and between location and length were not significant, both *F*’s < 0.6. Finally, the main effect of trial length was significant, *F*(1,26) = 6.388, *p* = .02, *η^2^_p_* = 0.197 (medium-to-large effect size), but the main effects of location and expertise were not significant, both *F*’s < 0.1.  As can been seen in Fig. 4, beta values were low for long trials in both the left and the right FFA for both laypeople and residents. In the short trials, beta values were higher for residents than for laypeople in the left FFA but beta values were higher for laypeople than for residents in the right FFA. Post-hoc t-tests show that none of those differences was significant, all *t’*s < 1.6.”  *Pages 20-21, lines 408-419:* “We also ran an exploratory 2x2x2 ANOVA with factors expertise level (laypeople and radiologists in training), trial length (2s trials and 10s trials), and location (left FFA and right FFA) on beta value as the dependent variable. The three-way interaction between expertise level, trial length and location showed a trend towards significance, *F*(1,26) = 3.937, *p* = .06, *η^2^_p_* = 0.132 (medium effect size). There was also a trend towards significance for the interaction between location and expertise level, *F(*1,26) = 2.885, *p* = .10, *η^2^_p_* = .100 (small-to-medium sized effect). The interactions between trial length and location and trial length and expertise level were not significant, both *F’*s < 0.7. The main effect of trial length was significant, *F*(1,26) = 10.91, *p* = .003, *η^2^_p_* = 0.296 (large effect). The main effects of location and expertise level were not significant, both *F*’s < 0.3. Figure 5 shows the same pattern of results as when all trials were included. Post-hoc t-tests show a trend towards significance for the right FFA short runs, *t*(26) = 1.80, *p* = .08, Cohen’s *d* = 0.71 (medium-to-large effect); for the other t-tests all t’s <1.0.” |
| 2. I don’t think it will affect the importance of the manuscript if the V1 data is linked to the FFA effect, it is better to be transparent so readers can make up their own minds. This is why performing correlations between V1 and FFA activity are interesting (same is true with the partial out method). As I mentioned previously, even if there is a link between V1 and FFA effects, this is still consistent with the expertise hypothesis: see Gauthier’s recent commentary on Kanwisher’s paper on the history of FFA research, and Lohse et al. (2016) on this link to face regions. If the face processing link is expertise based, it makes sense if there’s a link due to non-face expertise. You should report these as exploratory. | *In line with the comment and that of the other reviewer, we have added ANCOVAs to partial out the V1 activity. Since repeated measures ANCOVAs would not be possible (the covariate itself has repeated measurement points), we look at V1 activity as a covariate in analyses that investigate the main effect of expertise, on page 22, lines 453-457:*  “Finally, as requested by reviewers, we use ANCOVAs to analyze expertise differences in short-presentation and long-presentation trials, both in all trials and only correctly diagnosed trials, with activity in V1 (during short-presentation and long-presentation trials, both in all trials or only correctly diagnosed trials) as the covariate. Table 2 shows F- and p-values for the covariate and the main effect of expertise.  **Table 2. F- and p-values for the ANCOVAs**”  *We discuss the implications of our findings in the discussion on page 25-26 lines 507-521:* “The analyses of V1 activity provide an insight into the attention account of expertise effect, which holds that expertise effects in the FFA and other brain regions are simply the effect of greater attentional engagement with the objects of expertise [9, 18]. That is, an overall larger level of attention to objects of expertise by experts (because they are more interested in those stimuli) causes expertise effects not just in the FFA but also in other regions of the visual system, such as V1. Gauthier argues against this account, showing expertise effects in the FFA even with limited attention (e.g., when the object of expertise is irrelevant), and showing expertise effects even in the regional grey matter thickness of the FFA. To explore the attention account of expertise for our data, we investigated whether radiologists in training show larger V1 activation (as a result of larger attentional engagement) then novices, and found no evidence for this. Additionally, as suggested by reviewers, we investigated whether partialling out V1 activation removes expertise effects, and found indeed that differences between conditions were no longer significant after partialling out V1 activity. This means that we cannot exclude that increased attention for the stimuli by residents compared to laypeople explains the pattern of results in the FFA. On the other hand, the V1 activity is not a significant covariate in any of the ANCOVAs.” |
| 3. Can’t the authors ‘deface’ the raw fMRI data so they remove the risk of identification? Doesn’t this mean they could then make the data publically available? Also, I can’t access the data file the authors provide to replicate their analyses. | *The data file is now freely accessible on* [*https://doi.org/10.34894/O0CKVP*](https://doi.org/10.34894/O0CKVP)*, access to the raw data can be requested using the contact function in Dataverse.*  *As to sharing raw data, we comply with the ethical committee’s recommendation and the regulations of the data manager by not sharing the raw fMRI data. We would like to stress that interested researchers can easily request to receive the raw data files from the data manager through Dataverse. There is just one additional check in place to ensure that the participants and their colleagues cannot access the files. We agree that data sharing is important for open science, but we communicated to our participants that we would not share raw data openly, so we cannot change this for the current study. For follow-up studies, we will discuss the option of sharing raw data openly. As an explanation for the ethical committee’s recommendation here, we would like to mention that the ethical committee considered it particularly problematic to share raw data given that participants have software and knowledge required to interpret the MRIs and might even be able to identify colleagues in defaced MRIs.* |
| 4. The description in the Methods of how the authors decided their sample size still doesn’t appear to be based on any objective criteria. Saying you decided to test 10 in this group and 10 in another group is not the same as using power estimates from prior work to decide sample sizes. I am mindful that pushing this issue further may make a posthoc decision appear, but why were these numbers decided (the authors mention line numbers 168-170, but I don’t see this in my pdf, maybe they mean 147-149), was power ever considered when designing your study? The decision to stop at 17 experts for practical reasons is again too vague. What were these reasons? Funding spent elsewhere? Funding expiring? Scanner was no longer available to the researchers? You asked all your radiologists and the others said no? Or you stopped as you found a significant effect? It should be stated. | *In hindsight, we should have paid more attention to objective criteria to determine power when we set up the study, but we unfortunately cannot change that anymore. We agree that there is a risk of readers misinterpreting how the literature currently stands with respect to power considerations, and that it is important to be transparent to readers that determining the sample size in fMRI research such as this requires a shift in approach. Therefore, we now elaborate on our considerations in the Method section and added recommendations for sample size determination for future research in the Discussion.*  *We now write on pages 7-8 lines 159-166:* “An a-priori determined sample size of 10 residents in their first year and 10 residents in year 3 or higher was selected because this sample size seemed maximally feasible given the number of eligible participants at reasonable travelling distance from the fMRI facilities, and was in line with earlier studies such as [2, 4, 16, 25, 26]. However, given that fewer eligible participants than expected were willing to participate, in combination with limited availability of funding, the a-priori determined sample size was not met. Therefore, the two groups of residents were combined into one group (radiologists in training).”  *In the discussion, we elaborate on the sample size in more detail and encourage readers to increase sample sizes, page 30, lines 615-622:* “The issue of power in fMRI has received increasing attention [33], with suggestions to execute power analyses before data collection in a pilot study. This is still seldomly done and complex in expertise studies where eligible participant populations are small. In our study, two issues increased power: The blocked design and the sufficiently high number of trials. Unfortunately, our sample size, loosely based on those earlier findings, might have been too small given our current understanding of the size of the expertise effect. With this, our study gives tentative evidence that indeed expertise effects can be found in the right FFA in the holistic mode but less so in the search-to-find mode, but further research with larger samples is necessary to corroborate our findings.” |
| 5. The following points all touch upon issues of power that the authors discuss. The authors cite Gegenfurtner et al. in their discussion, which is a review of eye tracking effect sizes, but the current study is fMRI. I don’t think this is appropriate and should be removed as it reminds me of the Cow-Canary problem where effects can be massively different between two different methods (Capitani et al., 1999). The authors’ own behavioural data here shows a huge effect size (which would be expected between experts and novices), yet only a medium one in the fMRI data. Why discuss eye-tracking effect sizes when you had 20-30 fMRI expertise studies to base your decision on, it doesn’t make any sense in this context and should be removed as just confuses the issue.  In the same place in the Discussion the authors seem tacitly argue two fMRI expertise studies that are 20 years old are an acceptable guide for sample sizes (Gauthier et al., 1999: which they don’t cite here but describe, and Gauthier et al., 2000). This ignores the recent studies that used larger sample sizes (peaking with the recent Martens et al paper, which is not cited, nor most other studies in the last 10 years). Even if two 20 year old papers were the basis for making their sample size decisions, it should be mentioned in the Methods and not the Discussion (although again, a question remains as to why the authors selected such old papers to base their sample sizes on rather than more recent work).  From the above I hope it’s clear why the current Discussion paragraph on effect sizes from eye tracking and two 20 year old fMRI papers does not work. I don’t want future researchers reading this paper to think these sample sizes would lead to sufficient levels of power when designing their own fMRI expertise studies. I think it would be more helpful to explicitly remind the readers that we now know this study and Harley’s were likely underpowered, that classic papers from 20 years ago were underpowered, and that significant effects here were found using liberal alphas. It’s perfectly fine accepting the limitations of this manuscript, but they need explained to the readers so they’re aware of the issues. | *We understand the reviewer’s concern and agree that readers should not be given the impression that sample size as small as ours should be sufficient in expertise research nowadays, and that earlier studies might have overestimated the effect sizes. We now removed the paper by Gegenfurtner et al. as a reference and encourage readers to execute power analyses. Recent studies such as the Burns and the Martens paper were not published at the time of our sample size decisions.*  *We now write on pages 7-8 lines 159-166:* “An a-priori determined sample size of 10 residents in their first year and 10 residents in year 3 or higher was selected because this sample size seemed maximally feasible given the number of eligible participants at reasonable travelling distance from the fMRI facilities, and was in line with earlier studies such as [2, 4, 16, 25, 26]. However, given that fewer eligible participants than expected were willing to participate, in combination with limited availability of funding, the a-priori determined sample size was not met. Therefore, the two groups of residents were combined into one group (radiologists in training).”  *In the discussion, we now write on page 30, lines 615-622:* “Recently, it has been argued that those earlier studies on the expertise account of the FFA with small sample sizes have overestimated the expertise effects [15]. The issue of power in fMRI has received increasing attention [33], with suggestions to execute power analyses before data collection in a pilot study. This is still seldomly done and complex in expertise studies where eligible participant populations are small. In our study, two issues increased power: The blocked design and the sufficiently high number of trials. Unfortunately, our sample size, loosely based on those earlier findings, might have been too small given our current understanding of the size of the expertise effect. With this, our study gives tentative evidence that indeed expertise effects can be found in the right FFA in the holistic mode but less so in the search-to-find mode, but further research with larger samples is necessary to corroborate our findings.” |
| 6. Also related to the previous points. Maybe I was not clear enough in my first review, but when I referred to page 20 and the different patterns of results, I meant that you failed to find a correlation but Harley and colleagues did. However, you found a group experience difference that Harley didn’t. The simplest explanation for this disparity is that both studies are underpowered. If you have two studies that are underpowered (maybe 50ish%), then it’s likely one (Harley et al) will find a significant effect in one analysis, but not the other, while the second paper (the current manuscript) finds the opposite pattern of significant/non-significant results with the same analyses. You haven’t discussed this in this paragraph, nor in the later paragraph where you argue your study is not underpowered based on two studies from 20 years ago. You should explain this hypothesis of the conflicting results explicitly when discussing the differences between Harley and yourselves. I should add, the discussion of the 2s vs 10s trials is distinct from the point I’m trying to make here (although a similar logic could be applied, I think the correlation/group difference disparity needs explained separately). | *We now added the relatively low number of participants as a potential explanation for the difference between our study and that of Harley and colleagues on page 29 lines 608-613:*  “A final important limitation of our study is the limited number of participants. As a reviewer pointed out, this could also explain that we found the opposite pattern of results as Harley and colleagues [2].” |
| Minor points 1. The authors uploaded two different versions of their manuscript, one with tracked changes indicated and another without, but switched between them when they were referring in their response to reviewers (and they sometimes referred to line numbers that didn’t correspond with the text). This made checking between the different texts confusing and overly time consuming to review (and apologies if I’ve now referred to one text and the other). I’d recommend on the next submission they simply change the font color of the text they’d edited and upload a single manuscript (unless this is a journal specific requirement to upload two?). Same is true of having the figure legends next to the actual figures. | *We apologize for the inconvenience caused by the errors in the line numbers and double-checked the line numbers in this version.*  *As to your recommendations, the journal requires that two manuscripts are uploaded and the journal also requires that* “Figure captions must be inserted in the text of the manuscript, immediately following the paragraph in which the figure is first cited (read order). Do not include captions as part of the figure files themselves or submit them in a separate document.” |
| 2. I think the authors have misinterpreted signal detection theory by claiming they can’t compute dprime. They employed a 2AFC task, with stimulus pairs that were same or different. This leads to categories of hits, misses, CRs and FAs. It’s quite easy to compute dprime to test whether this behavioural measure is more strongly correlated to FFA activity as it was in Harley et al. I’m convinced other readers would want to see this to compare the results between the current study and Harley et al. | *In this study, we did not employ a 2-AFC task. During a 2-AFC experiment, a subject makes a decision between two stimuli about a stimulus parameter of interest while in our study, only one stimulus was presented at the time (see* [*https://www.sensorysociety.org/knowledge/sspwiki/Pages/Alternative%20forced%20choice.aspx*](https://www.sensorysociety.org/knowledge/sspwiki/Pages/Alternative%20forced%20choice.aspx)*).*  *Signal detection theory could indeed still be applied in our study and d-prime can be calculated. Our dependent measure of diagnostic accuracy is calculated as Hitrate + (1- false alarm rate), whereas d-prime is calculated as (zhitrate - zfalsealarmrate). Those are almost equal and thus correlate highly (r = .99) so we cannot report both.*  *We choose not to report our measure as d-prime, as we think it is not comparable with other d-prime measures, such as the one in Harley et al. Harley’s d-prime measures the perceptual process of detecting the abnormality (abnormality present/absent). Since potential abnormalities were cued in their study, only the ‘decision’ phase of the perceptual process was measured. Our measure ‘diagnostic accuracy’ is not a perceptual measure (because participants know there is an abnormality), but a cognitive measure (which abnormality is this?). We thus believe that our d-prime and Harley’s d-prime reflect different phenomena. Therefore, we think that by reporting d-prime instead of diagnostic accuracy, we might confuse the reader.* |
| 3. Line 84: “processing a face as a whole, and not as a combination of features” I think referring to ‘combination of features’ implies a holistic relational percept. Maybe something like “as separate, distinct features that do not interact to form a single percept” is clearer? | *We now write on page 4 lines 90-91:* “processing a face as a whole, and not as set of separate, distinct features that do not interact to form a single percept.”. |
| 4. Removing the original graphs stops making the group differences/similarities in the different conditions easily comparable so should be reinstated. I like the individual plots, but trendlines would be a useful addition too. | *We have now included 4 new figures (figures 4,5,6,7) that show both the group differences as well as individual data points (scatterplots) for both the FFA and the V1 activity.* |
| 5. Line 490 still reads like prior studies analysed an area that included the FFA (i.e., you mention overlap), but we can’t say this as they did not localise it. It could be in the vicinity of the FFA, but not actually overlap . | *We now write on page 27 lines 556-559 that “*Neither Haller and Radue [25], nor Melo and colleagues [26] found areas with significant activation in the vicinity of the FFA. Note that they did not employ an FFA localizer task but only report areas with significant activation.” |
| Related to this, there are many expertise FFA studies that did localise the FFA and are not cited in this manuscript which seem more relevant (particularly the Martens et al study which should be the gold standard going forward). Surely at least from a point of generating interest in the current paper the authors would want to reference these as more citations lead to more exposure? | *We now discuss a larger range of papers that found expertise effects in the FFA, including the Martens et al paper page 5 lines 72-81:*  “After the classic studies of Gauthier and colleagues, expertise effects in the right FFA were established in a large number of studies [15] with various objects of expertise [15], such as cars, [18-20], birds and minerals [21], and butterflies and moths [22]. Since it has been argued that those objects have a face or face-like structure [11], other investigations focused on less face-like objects such as chess-boards [3, 14]. Again, expertise-related activation in the right FFA was found. For example, Bilalíc and colleagues To further strengthen the argument, Bilalić and colleagues investigated the effect of expertise on FFA activation in stimuli with less face-like features: chess boards [3]. and chest radiographs [23]. They found that the FFA is differently engaged in experts versus novices when chess positions were presented, but not when single chess pieces were presented [3].”  *We also refer to the Martens et al study as an example of a study that investigated the involvement of other brain regions in visual expertise on page 28, line 588-590*: “it would be interesting to also investigate the involvement of other regions of the brain in other aspects of visual expertise, such as areas in the occipital and frontal cortex, see e.g., [21]” |
| Reviewer #2: |  |
| I appreciate the authors modified and responded to each of the points I raised in the review. My remaining concern for the paper is obviously the insignificance in the difference of rFFA activation between experts and laypeople for 2s trials. The statistics showed that for all trials, Ttest p=0.06, for correct trials ttest p=0.04, ANOVA results for expertise vs laypeople are all insignificant. Such small effect could be caused by the two dots shown in fig 4 and 5, which showed around 2 and 3 in beta values, but their diagnostic performance were low, and the training background were short. Given all the main conclusions and novelties for this paper are based on this small “trend”, and there seems no way the authors could recruit more subjects and alter that, I therefore am not quite confident to believe what have been concluded in the paper.  Reviewer #1 pointed out a paper by Burns, Arnold and Bukach 2019 in Neuroscience and biobehavioral reviews, in which the authors tried to justify p-values close to 0.05 could still be significant by meta-analysis. I am however not convinced that such meta-analysis on different tasks could justify the current small “trend” seen in the paper under review. | *We have adapted the discussion to provide a more nuanced discussion of our findings.*  *Page 26 line 523* “In comparison, we found tentative evidence that the right FFA already responds to expertise-related stimuli in radiologists in training with only 1-5 years of experience in radiology” *(underlined words new).*  *And page 29 lines 620-622* “With this, our study gives tentative evidence that indeed expertise effects can be found in the right FFA in the holistic mode but less so in the search-to-find mode, but further research with larger samples is necessary to corroborate our findings.”  *And page 30 line 629* “In conclusion, our data provides tentative support for the general expertise hypothesis of FFA functioning in a group of radiologists in training.” |
| I also don’t quite like the author removed the V1 results. Like reviewer #1 suggested, I think the authors should use V1 to normalize the rFFA response, to control the possible attention/engagement bias in both subject groups and expertise levels. Literatures have shown that early visual areas including LGN, V1 and V4 all show attentional modulations in fMRI studies. | *We have now reinstated those analyses, see an overview of all changes in response to reviewer 1.* |
