# Supplementary material for: Holistic processing only? The role of the right fusiform face area in radiological expertise
Source: PLoS One. 2021 Sep 1;16(9):e0256849. doi: 10.1371/journal.pone.0256849 (PMC8409609; doi:10.1371/journal.pone.0256849)
Supplement: Attachment — Submitted filename: ReponsetothereviewersR3.docx [file pone.0256849.s004.docx]

Dear editor,

Thank you for providing us with the opportunity to revise the manuscript. Below, we detail how we have adapted the manuscript in response to the comments of the reviewers. Additionally, the reference list was checked for completeness and correctness.

Kind regards on behalf of all authors,

Ellen Kok

1. *In the previous drafts, the authors ran a one-tailed t-test on the right FFA short run which showed an effect of expertise, but for some reason they are no longer reporting this as one-tailed, but two-tailed now on line 401 (I think, the t-value is identical and the p-value is now double what it was previously in the deleted section), which renders the effect of expertise non-significant. I can't work out why this has been changed? I think it would be beneficial if this was changed back to the one-tailed test as that was what the authors initially intended and corroborates what they're actually claiming, i.e., an effect of expertise in the right FFA. There may need to be some minor text adjustments because of this (e.g., paragraph at line 466).*

We had adapted the analysis to a two-sided test because all other tests were two-sided. Since we originally had a one-sided test (because we had a specific hypothesis, we have now changed the sentence to read:

*“Post-hoc t-tests show a significant effect for the right FFA short runs, t(26) = 1.80, p = .04 (one-sided t-test), Cohen’s d = 0.71 (medium-to-large effect); for the other t-tests all t’s <1.0.” (lines 401-402 in the version without tracked changes).*

Additionally, we changed ‘trend towards significance for the difference’ to ‘significant difference’ in line 471 in the version without tracked changes.

2. *Lines 481-483 need citation/s.*

We have now added the citation that was accidentally left out for line 481-482 (reference 35).

3. *There were a couple minor typos so a proofread may be good (e.g., line 77 gap before comma).*

An additional thorough proofread was conducted, fixing the typo in line 77 and several others.
